# Profiles of Aggressiveness and Stress in Spanish Adolescents

**DOI:** 10.3390/ejihpe15060112

**Published:** 2025-06-13

**Authors:** Cecilia Ruiz-Esteban, Inmaculada Méndez, Juan Pedro Martínez-Ramón, Nuria Antón-Ros, Nelly Gromiria Lagos San Martín

**Affiliations:** 1 Department of Developmental Psychology and Education, University of Murcia, 30100 Murcia, Spain; cruiz@um.es (C.R.-E.); juanpedromartinezramon@um.es (J.P.M.-R.); 2 Department of Developmental Psychology and Didactics, University of Alicante, 03080 Alicante, Spain; nuria.anton@ua.es; 3 Educational Science Department, University of Bío-Bío, Chillán 3780000, Chile; nlagos@ubiobio.cl

**Keywords:** aggressive behavior, educational psychology, latent profile analysis, school stress, secondary education

## Abstract

Aggressiveness among schoolchildren can be shaped by specific school-related situations that elicit stress. Accordingly, this study aimed to identify differentiated profiles of secondary school students based on the levels of aggressive behavior they exhibit. A further objective was to examine whether various stress-related factors differ significantly across these behavioral profiles. The sample consisted of 386 secondary school students (*M* = 13.73; *SD* = 1.14), of whom 52.6% were female. Data were collected using the Aggression Questionnaire (AQ) by Buss and Perry and the School Situation Survey (SSS) developed by Helms and Gable. Latent profile analysis revealed three distinct profiles: (a) students exhibiting high levels of aggressive behavior (Cluster 1), (b) students showing moderate levels of aggressive behavior (Cluster 2), and (c) students displaying low levels of aggressive behavior (Cluster 3). Students in the high-aggression profile reported significantly higher scores on most sources of stress compared to their peers in the moderate and low aggression profiles. From an educational standpoint, these findings underscore the importance of addressing school-related stressors, as they appear to play a critical role in influencing student behavior.

## 1. Introduction

Aggressiveness is a multidimensional psychological construct encompassing cognitive, emotional, and behavioral components (Buss & Perry, 1992). Cognitively, it involves perceptions of injustice, attributions of hostile intent—a process aligned with Berkowitz’s (2012) emphasis on aversive stimuli activating aggressive cognitive schemas—and beliefs legitimizing harm (Crick & Dodge, 1994). Emotionally, it manifests as anger, a transient affective state ranging from irritation to rage, which Berkowitz (2012) theorized as a critical mediator between negative experiences and aggressive impulses, alongside physiological arousal, such as increased heart rate or cortisol levels (Koyama et al., 2024; Gunnar & Quevedo, 2007). Behaviorally, it includes overt actions like physical or verbal aggression (e.g., hitting, insults), covert acts such as relational aggression (e.g., social exclusion), and increasingly pervasive digital forms like cyberbullying (García-Sancho et al., 2016; Van Wert et al., 2016; Kasturiratna et al., 2025). Buss and Perry’s (1992) framework further delineates four instrumental subdomains: physical aggression (direct bodily harm), verbal aggression (harmful speech), anger (emotional reactivity), and hostility (cognitive resentment). This multidimensionality underscores its complexity, necessitating holistic assessment tools, such as the Aggression Questionnaire (AQ), validated across diverse populations (Torregrosa et al., 2020; Santisteban et al., 2007).

Adolescence, marked by neurobiological maturation and profound psychosocial transitions, represents a critical period for studying aggression. Hormonal changes, particularly in testosterone and cortisol, amplify emotional reactivity and impulsivity (Arnett, 1999), while concurrent cognitive restructuring and the refinement of socio-emotional competencies shape how adolescents appraise and respond to stressors (Crone & Dahl, 2012; Steinberg, 2007). Social reorientation—such as heightened peer influence, identity formation, and immersion in digital environments—further heightens sensitivity to environmental demands (Compas et al., 2001; Kasturiratna et al., 2025). Schools, as microcosms of social interaction, expose adolescents to stressors such as academic competition, peer conflict, authority dynamics, and cybervictimization (Kasturiratna et al., 2025; Lazarus & Folkman, 1984). For instance, perceived teacher bias, academic failure, or online harassment can trigger frustration, activate hostile cognitive schemas (Espelage & Swearer, 2003) and reinforce maladaptive social-information processing patterns, such as hostile attribution biases (Dodge, 2006). Conversely, supportive environments may mitigate aggression by fostering resilience through improved emotion regulation strategies (Selye, 1950; Yeager et al., 2018), aligning with Berkowitz’s (2012) acknowledgment of cognitive reappraisal as a moderating factor. However, chronic stress, as seen in bullying or cyberbullying victims, often dysregulates both emotional control and executive functioning, exacerbating impulsive or preemptive aggressive responses (Kasturiratna et al., 2025; Olweus, 1993; Oliveira et al., 2020). Cognitive advancements in theory of mind, while enhancing perspective-taking under normal conditions, may paradoxically amplify perceived social threats in high-stress contexts, such as peer exclusion or online disinhibition (Kasturiratna et al., 2025; Zimmer-Gembeck & Skinner, 2011). Thus, adolescence represents a nexus where biological predispositions, environmental demands, digital interactions, and developmental shifts in cognition—such as risk–reward evaluation and moral reasoning—interact to shape aggressive trajectories.

Empirical research has consistently validated the four-factor aggression model (anger, hostility, verbal aggression, and physical aggression) across developmental stages. Torregrosa et al. (2020) confirmed this structure in Spanish children using the Aggression Questionnaire–Short Form (AQ-SF), identifying five aggression profiles linked to school anxiety. Cross-cultural studies reveal significant gender differences. This was corroborated by Santisteban et al. (2007) in Spanish adolescents, where male physical aggression and female hostility were linked to distinct psychosocial factors. These findings reflect both socialization influences (e.g., emotionally restrictive expression in males versus conflict internalization in females) and potential neurocognitive underpinnings (Archer, 2004). Contextual stressors—such as academic pressure, cyberbullying, or peer victimization—modulate these aggressive expressions in gender-specific ways. For instance, Oliveira et al. (2020) demonstrated that bullying victimization correlates with suicidal ideation in females but predicts reactive aggression in males. Recent meta-analyses emphasize that cyberbullying disproportionately affects females and marginalized groups, exacerbating internalizing symptoms (anxiety, academic disengagement) and retaliatory aggression (Kasturiratna et al., 2025). Academic stress—defined as perceived inadequacy toward scholastic demands (Depraect et al., 2017)—intensifies hostility in females and physical aggression in males, particularly when coupled with ineffective coping strategies (Acosta & Rúa, 2021). Physiological markers like elevated cortisol further impair prefrontal regulation of impulsive behaviors (Gunnar & Quevedo, 2007), creating a bio-behavioral substrate that interacts with gender factors to shape aggressive trajectories in educational settings. Despite progress, critical gaps limit current understanding. Theoretically, most frameworks prioritize individual traits over systemic factors and digital dynamics. For example, while Buss and Perry’s model delineates aggression components, it neglects how school-specific stressors (e.g., teacher–student rapport, cyberbullying exposure) differentially activate these dimensions. Methodologically, studies often rely on cross-sectional designs or self-reports, overlooking temporal dynamics, physiological correlates (e.g., cortisol assays), and digital behavioral data. Contextually, research focuses on Western populations, limiting insights into cultural variability—a concern given that collectivist societies may suppress overt aggression differently (Morales-Vives et al., 2005). Additionally, few studies integrate stress resilience into aggression models, despite Selye’s (1950) emphasis on adaptive coping. For instance, Di Fabio and Bucci (2015) linked poor stress adaptation to self-destructive behaviors but did not explore protective factors (e.g., extracurricular engagement, digital literacy programs). Finally, while tools like the AQ-SF are validated for efficiency (Morales-Vives et al., 2005), their application in secondary education—a period of escalating academic, social, and digital demands—remains underexplored.

This study addresses these gaps by examining aggression profiles in secondary students through a systemic lens. First, it employs the AQ-SF to classify adolescents into subgroups (e.g., high-hostility vs. high physical aggression), extending Torregrosa et al.’s (2020) work to an older cohort. Second, it evaluates how distinct school stressors—teacher interactions, academic pressure, peer dynamics, and cybervictimization—interact with these profiles, a novel approach that moves beyond singular stressor analyses (e.g., Kasturiratna et al., 2025; Oliveira et al., 2020). Third, it integrates emotional, behavioral, digital, and physiological stress indicators, addressing Compas et al. (2001) call for multidimensional assessment. By doing so, this study highlights contextual triggers of aggression, such as whether teacher-related stress exacerbates hostility more than peer conflict or cyberbullying. Practically, these insights can inform tiered interventions: for example, training teachers to de-escalate hostility, designing peer mediation programs for verbal aggression, or implementing anti-cyberbullying policies. Theoretically, it advances ecological models of aggression by situating individual traits within institutional and digital dynamics, bridging a critical gap in educational psychology literature.

By synthesizing developmental, contextual, digital, and stress-related perspectives, this study offers a nuanced understanding of adolescent aggression. Its findings hold implications for fostering adaptive school climates and safer online environments, ultimately contributing to the broader goal of enhancing student well-being and academic success.

## 2. Materials and Methods

### 2.1. Design and Procedure

Initially, approval was obtained from the Research Ethics Committee of the University of Murcia (ID: 3749/2022). Following this, the research team presented and discussed the objectives of the study with the management teams of the educational institutions from where the sample was selected. Data collection proceeded after obtaining informed consent from the parents and informed assent from the minors. The questionnaires were administered in person, using paper-based formats, during a 45 to 50 min session, and were individually distributed to each classroom group within the schools. Throughout the process, confidentiality of the data, voluntary participation, and anonymity were ensured. The data were then entered into a database and subjected to statistical analysis. The research was carried out in the year 2024.

### 2.2. Participants

The study involved 386 adolescents aged between 12 and 15 years (*M* = 13.73, *SD* = 1.14), recruited from five randomly selected schools—four public and one private—located within an autonomous community in Spain. The sample was obtained using a cluster sampling approach. The primary sampling units consisted of five geographic regions selected from a southeastern Spanish province (north, south, center, east, and west), encompassing both public and private schools across rural and urban settings. Secondary units comprised classrooms: four classrooms were randomly selected per school, with one classroom representing each grade level. School size determined the number of class groups per grade, ranging from one to three groups, which translated to an estimated student population of 30 to 90 per school. The sample was evenly distributed in terms of age and sex (*χ*^2^ = 2.57, *p* = 0.50), comprising 47.4% male and 52.6% female participants. Socioeconomic background was assessed through parental education levels: 10.7% of fathers and 14.2% of mothers had completed primary education; 61.19% of fathers and 66.55% of mothers had attained secondary education; and 17.05% of fathers and 11.31% of mothers held university degrees. Information on family structure indicated that 65% of participants lived with married parents, 14.2% lived with divorced or separated parents, and 20.8% lived in other family arrangements (e.g., cohabiting, single-parent households). In terms of sibling status, 14.7% of the adolescents were only children, 57.5% had one sibling, 18.2% had two siblings, and 8.6% had three or more siblings. Cases with missing data on parental education were excluded from the relevant analyses (Table 1).

### 2.3. Instruments

To assess aggressive behaviors among students, the Aggression Questionnaire (AQ) developed by Buss and Perry (1992) was used in its Spanish-validated version by Santisteban and Alvarado (2009). This self-report instrument consists of 29 items designed to measure aggressive behaviors and feelings. Participants assessed each statement on a Likert scale, where 1 represented “completely false” and 5 represented “completely true for me”. The questionnaire was structured around four factors, each demonstrating adequate reliability within the instrument, with Cronbach’s alpha values falling within the recommended thresholds: physical aggression (α = 0.82), which refers to aggression involving the use of one’s body or an external object to inflict harm; verbal aggression (α = 0.74), which pertains to the use of insults, rumors, threats, etc., to harm others; anger (α = 0.72), which represents the emotional response triggered by preceding hostile attitudes; and hostility (α = 0.77), which involves negative feelings and cognitive evaluations directed toward others.

Secondly, to evaluate stress factors among students, the School Situation Survey (SSS) developed by Helms and Gable (1989) was administered. This survey is designed for students aged 9 to 19 and is aimed at identifying students’ perceptions of school-related situations that may induce stress. The instrument comprised 34 items, each with 5 response options, ranging from 1 (never) to 5 (always). The scale is divided into seven subscales, each demonstrating adequate internal consistency, as indicated by Cronbach’s alpha values: teacher interaction (α = 0.70), which assesses students’ perceptions of teachers’ attitudes toward them; academic stress (α = 0.72), which evaluates the stress students experience in academic evaluation situations and its impact on their performance; peer interactions (α = 0.73), which measures the quality of students’ interactions with peers and their self-perception in academic contexts (α = 0.72), which assesses aspects related to students’ self-evaluation of their academic abilities; emotional stress (α = 0.78), which gauges feelings of shyness, sadness, loneliness, and similar emotions; behavioral stress (α = 0.70), which evaluates observable manifestations of anxiety, such as reactions and behaviors; and physiological stress (α = 0.71), which refers to physiological responses such as trembling, nausea, and others. Higher scores on each subscale indicate greater perceived stress in the respective domain.

### 2.4. Data Analysis

To address the objectives of the present study and classify participants into distinct profiles, a latent profile analysis (LPA) was conducted. The methodology employs a case-centered approach, identifying latent profiles by analyzing similarities and differences across cases through the estimation of means, variances, and covariances for each derived profile (Tein et al., 2013). To determine the optimal profile solution, Wang and Wang (2012) advocate for two key criteria: first, lower values in information-based indices, specifically the Akaike Information Criterion (AIC) and Bayesian Information Criterion (BIC), which indicate improved model fit; and second, statistically significant likelihood ratio tests, including the Vuong–Lo–Mendell–Rubin likelihood ratio test (LRT) and the Bootstrap Likelihood Ratio Test (BLRT), where *p*-values must fall below 0.05 to confirm the adequacy of the selected profile structure. This dual emphasis on statistical parsimony and empirical validation ensures robustness in classifying latent subgroups. The use of this person-centered approach was particularly appropriate, as it enables the identification of qualitatively distinct subpopulations based on adolescents’ patterns of aggressiveness and stress. Unlike variable-centered methods, which may overlook individual variability by focusing on overall associations between variables, LPA can uncover latent configurations that remain hidden in traditional analyses. This is especially relevant in the context of adolescent development, where complex interactions between personal, contextual, and digital factors may manifest differently across individuals. Thus, the application of LPA contributes to a more nuanced and ecologically valid understanding of aggression profiles, supporting targeted interventions within educational and online settings. Following the selection of the optimal aggressive behavior profile model, multivariate analyses of variance (MANOVA) were conducted to examine the differences in stress manifestations across the aggression profiles. Partial eta squared (*ηp*^2^) values and Cohen’s d were calculated to assess the magnitude of these differences, with the Bonferroni correction adjusted post-hoc comparisons applied to account for multiple testing. The effect size of observed differences was assessed using Cohen’s d (Cohen, 1988), interpreted as follows: small magnitude (*d* = 0.20–0.49), moderate magnitude (*d* = 0.50–0.79), and large magnitude (*d* ≥ 0.80). Statistical analyses were carried out using SPSS (Version 24.0) (IBM Corp, 2019) and the MPlus software package (version 8.4).

## 3. Results

The analyses conducted in this study identified three latent profiles of aggressiveness among secondary education students, based on the levels of aggressive behaviors reported. As shown in Table 2, the three-profile model was selected as the optimal solution, demonstrating the lowest values for both the Akaike Information Criterion (AIC) and Bayesian Information Criterion (BIC), along with the highest entropy. These results indicated superior classification clarity and model parsimony. Models with four to seven clusters were discarded due to non-significant likelihood ratio tests (LRTs) and the presence of clusters comprising less than 1% of the sample. Among the remaining models, the three-cluster solution provided the best statistical fit and interpretability, balancing theoretical coherence with empirical robustness. This model was retained for further profile characterization and analysis.

**Figure 1:**
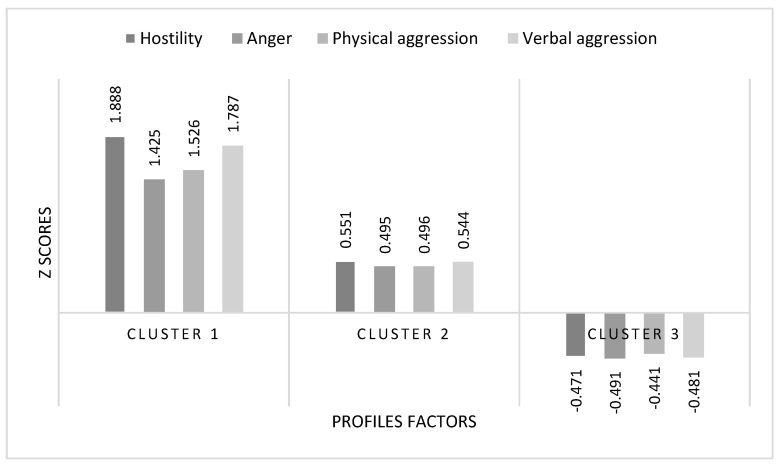
Graphical representation of the three-profile model with standardized (z) scores.

This model classified the students into three profiles: (a) a group of 37 students (9.6%) who exhibited high levels of aggressive behaviors (Profile 1), (b) a group of 156 students (40.4%) with intermediate levels of aggressive behaviors (Profile 2), and (c) a group of 193 students (50%) with low levels of aggressive behaviors (Profile 3). Figure 1 provides a graphical representation of the distribution across these three profiles.

Gender distribution within each profile was homogeneous (*χ*^2^ = 4.07, *p* = 0.13), demonstrating that the proportion of boys and girls did not differ significantly within individual profiles. This homogeneity suggests balanced gender representation across all subgroups when analyzed internally (Table 3).

The multivariate analysis of variance (MANOVA) revealed statistically significant differences in all stress variables when comparing the three aggression profiles (Wilks’ Lambda = 0.73, *F*(14,383) = 9.20; *p* < 0.001, *ηp*^2^ = 0.15), as detailed in Table 4.

The post hoc contrasts, adjusted using the Bonferroni method, revealed that students in Profile 1 (high aggression) had significantly higher scores in all stress dimensions compared to students in Profile 3 (low aggression). Additionally, in certain dimensions, such as teacher interactions and behavioral stress, students in Profile 1 also showed higher scores than those in Profile 2 (intermediate aggression). Conversely, students in Profile 2 scored higher than those in Profile 3 in all stress dimensions, with significant differences observed in academic stress.

In particular, the most notable differences were observed in the dimensions of emotional and behavioral stress, where students in Profile 1 showed significantly higher scores compared to those in the other two profiles. Cohen’s d indices (Table 5) indicated that the differences between the profiles were of moderate-to-large magnitude in most dimensions, particularly in emotional stress (*d* = 1.29 between Profile 1 and Profile 3) and behavioral stress (*d* = 1.45 between Profile 1 and Profile 3). These differences suggested that students with higher levels of aggression experienced greater emotional and behavioral distress in the school environment, which may have been related to a reduced ability to manage stress effectively.

Regarding academic stress, the post hoc contrasts, adjusted using the Bonferroni method, revealed that students in Profile 1 (high aggression) scored significantly higher than those in Profile 3 (low aggression). Similarly, Profile 2 (intermediate aggression) showed higher values than Profile 3. However, no significant differences were found between Profiles 1 and 2.

Concerning peer interactions, the post hoc contrasts showed that students in Profile 1 scored higher than those in Profile 3. Likewise, Profile 2 obtained higher values than Profile 3. No significant differences were observed between Profiles 1 and 2.

Regarding academic self-concept, the post hoc analyses showed that students in Profile 1 had higher scores than those in Profile 3. Similarly, Profile 2 scored higher values than Profile 3. No significant differences were detected between Profiles 1 and 2.

In the dimension of emotional stress, the post hoc contrast indicated that students in Profile 1 scored higher than those in Profile 3. Similarly, Profile 2 showed higher values than Profile 3. No significant differences were found between Profiles 1 and 2.

Regarding behavioral stress, the post hoc contrasts revealed that students in Profile 1 scored significantly higher than those in Profiles 2 and 3. Additionally, Profile 2 showed higher values than Profile 3.

Finally, in the dimension of physiological stress, the post hoc contrasts indicated that students in Profile 1 scored higher than those in Profile 3. Similarly, Profile 2 showed higher values than Profile 3. No significant differences were observed between Profile 1 and 2.

Therefore, students with higher levels of aggression (Profile 1) tended to experience higher levels of stress across various dimensions, while those with low levels of aggression (Profile 3) showed less impact in these areas.

In summary, the results confirmed that students with high levels of aggression (Profile 1) exhibited higher levels of stress in all evaluated dimensions, covering teacher interactions, academic pressure, peer relationships, academic self-concept, emotional stress, behavioral stress, and physiological stress. Conversely, students with low levels of aggression (Profile 3) showed the lowest levels of stress across all dimensions, suggesting a greater ability to adaptively cope with school-related situations. These findings underscore the importance of considering both individual and contextual factors in the study of aggression and stress in educational settings.

## 4. Discussion

In line with previous research (Armengol Ortiz & Sanmartín López, 2024; Buss & Perry, 1992; Torregrosa et al., 2020), the results of this study confirm the existence of distinct aggression profiles among secondary education students, supporting the notion that aggression is not a homogeneous construct but rather manifests in varying degrees and forms, associated with both individual and contextual factors. These findings resonate with Berkowitz’s Cognitive Neoassociation Theory, which posits that aggression arises from a network of cognitive and affective associations activated by aversive experiences, such as stress or perceived threats (Berkowitz, 2012). The identification of primary profiles aligns with Berkowitz’s emphasis on individual differences in how negative affect activates hostile schemas. While prior studies have identified four main factors, our findings group these four factors into three primary profiles: one characterized by high levels of aggression (Profile 1), another with intermediate levels (Profile 2), and a third with low levels of aggression (Profile 3). These results align with the literature suggesting that aggression in children can vary significantly based on cognitive, emotional, and behavioral factors (González-Peña et al., 2013).

Furthermore, the results indicate that students with higher levels of aggression (Profile 1) report significantly higher levels of stress across all evaluated dimensions, encompassing teacher–student interactions, academic pressure, peer relationships, self-concept in academic contexts, emotional stress, behavioral strain, and physiological stress. This suggests that more aggressive students are more likely to experience distress in the school environment, potentially linked to a more negative perception of school situations and likely diminished ability to manage stress effectively (Compas et al., 2001; Kaplan et al., 2005). These results are in agreement with previous studies demonstrating a bidirectional association between aggression and stress, where stress can co-occur with aggressive behaviors, and aggression is related to heightened stress vulnerability (Espelage & Swearer, 2003; Gunnar & Quevedo, 2007). Similarly, the current results align with findings by Oliveira et al. (2020), Acosta and Rúa (2021), and Trinidad (2021), which have indicated that higher levels of bullying are associated with increased stress.

In particular, students in Profile 1 exhibited significantly higher levels of stress in teacher interactions and behavioral stress compared to students in Profile 2. This may reflect relationships where more aggressive students experience greater challenges managing teacher expectations and demands, potentially correlating with a cycle of negative interactions that co-occurs with stress and aggression (Olweus, 1993). Additionally, the higher levels of emotional and physiological stress observed in Profile 1 students suggest a connection between heightened emotional and physiological arousal and aggressive behavior (Depraect et al., 2017; Lazarus & Folkman, 1984). On the other hand, students in Profile 3, who exhibited the lowest levels of aggression, also showed the lowest levels of stress across all evaluated dimensions. This indicates an association between lower aggression and adaptative stress management, potentially tied to emotional resilience and social adjustment (Compas et al., 2001). These findings align with research suggesting that students with low levels of aggression tend demonstrate strong coping skills and emotion regulation (Gunnar & Quevedo, 2007).

Our analysis revealed that gender distribution within each profile remained homogeneous, indicating no statistically significant differences in the proportion of boys and girls across individual profiles. This finding contrasts with claims by some authors suggesting gender-based disparities in aggressiveness. However, it is essential to contextualize these results considering cross-cultural studies that do reveal significant differences in gender-specific expressions of aggressiveness. For instance, Santisteban et al. (2007) observed in Spanish adolescents that physical aggression in males and hostility in females are linked to distinct psychosocial factors, reflecting both sociocultural influences (e.g., emotional restriction in males vs. internalization of conflicts in females) and neurocognitive bases (Archer, 2004). Furthermore, contextual factors—such as academic pressure, cyberbullying, or peer victimization—modulate these expressions in gender-specific ways. Oliveira et al. (2020), for example, demonstrated that bullying victimization correlates with suicidal ideation in females but predicts reactive aggression in males. These nuances suggest that, while our sample showed no structural differences in gender representation across profiles, associated behavioral and emotional manifestations may vary depending on context and individual experiences. The internal consistency observed in our subgroups reflects balanced representation in terms of proportion but does not rule out the possibility that underlying mechanisms—social, cognitive, or environmental—operate differentially. Although our data refute gender-specific variations in profile composition, they do not contradict evidence that associated expressions may be mediated by complex sociocultural and environmental factors.

The outcomes of this research have considerable relevance for educational practice. First, it is crucial for educators and school psychology professionals to recognize that aggression varies in form and is linked to individual student characteristics. This underscores the need for personalized interventions that target the cognitive, emotional, and behavioral factors associated with aggression (Torregrosa et al., 2020).

Second, the findings emphasize the importance of addressing sources of stress within the school environment, as these factors are correlated with aggressive behaviors. Interventions should focus on enhancing student–teacher interactions, reducing academic stress, and fostering a positive school climate that supports students’ emotional well-being (Espelage & Swearer, 2003). Emotional education programs aimed at enhancing regulation skills may also reduce aggression and its related emotional distress (Compas et al., 2001).

Additionally, students in Profile 1 might benefit from interventions addressing both aggressive behaviors and co-occurring stressors. These could include cognitive–behavioral strategies to modify negative perceptions and social skills training to improve peer/teacher interactions (Kaplan et al., 2005).

Furthermore, recent research underscores the critical role of unregulated environments, both digital and physical, in exacerbating adolescent aggression and adverse psychosocial outcomes, necessitating a broader reconceptualization of intervention targets. As highlighted by Kasturiratna et al. (2025), the absence of structured oversight in online spaces—such as social media platforms with lax content moderation—and offline contexts, including neighborhoods with limited community engagement, fosters environments where normative boundaries dissolve, enabling antisocial behaviors to proliferate. Concurrently, insufficient adult supervision deprives adolescents of protective scaffolding to navigate peer conflicts or toxic interactions, amplifying risks of aggression and emotional dysregulation. These findings challenge intervention frameworks narrowly focused on individual-level factors, urging policymakers and practitioners to prioritize systemic reforms. Strengthening regulatory mechanisms in digital ecosystems, enhancing community-based supervision programs, and fostering adult–youth mentorship networks emerge as imperative strategies to mitigate environmental stressors. By addressing these structural gaps, interventions can more effectively disrupt pathways linking disordered environments to maladaptive outcomes, thereby advancing holistic adolescent well-being.

Although this study offers important perspectives on the connection between aggression and stress in the school context, it is important to acknowledge some limitations. While this study highlights stress–aggression pathways, it underlines Berkowitz’s (2012) call to explore individual–context interactions. This cross-sectional design precludes causal conclusions; longitudinal research is needed to explore temporal associations between aggression and stress. The sample’s restriction to Spanish secondary students limits generalizability; replication in diverse cultural/educational contexts is warranted. Replicating this study in different populations would help confirm the validity of the findings.

Additionally, future research could explore the role of other factors, such as social support, family climate, and coping skills, in the relationship between aggression and stress (Pérez-Fuentes et al., 2019). It would also be valuable to investigate the impact of specific interventions aimed at reducing aggression and stress within the school environment, to identify the most effective strategies for promoting students’ emotional and social well-being. Future research should explicitly examine gendered mediation mechanisms—such as how socialization practices shape stress appraisal—to refine gender-responsive interventions (Archer, 2004; Zimmer-Gembeck & Skinner, 2011).

## 5. Conclusions

This study confirms the existence of distinct aggression profiles among secondary education students and emphasizes the importance of addressing stressors in the school environment to mitigate aggressive behaviors. The findings suggest that students with higher levels of aggression experience elevated stress across multiple dimensions, highlighting the necessity for personalized interventions targeting both aggression and stress. Future research should further investigate this relationship to develop more effective strategies to foster a positive school environment and decrease the prevalence of disruptive behaviors.

## Figures and Tables

**Table 1:** Sample distribution.

%	Parental Education Level	Sex	
		Men	Female
Primary Education		10.7	14.2
Secondary Education		61.19	66.55
University		17.05	11.31
Total		47.4	52.6
%	Family structure		
Married		65	
Divorced or separated		14.2	
Other family arrangement		20.8	
%	Sibling status		
Only children		14.7	
One sibling		57.5	
Two siblings		18.2	
Three or more siblings		8.6	

**Table 2:** Fit indices for the latent profile models.

Models	AIC	BIC	BIC-Adjusted	LRT *p*	LRT-Adjusted	BLRT	Entropy	Size Group
2	3541.91	3593.33	3552.09	<0.001	<0.001	<0.001	0.83	0
**3**	**3342.07**	**3413.27**	**3356.16**	**<0.001**	**<0.001**	**<0.001**	**0.87**	**0**
4	3311.07	3402.05	3329.08	0.305	0.315	<0.001	0.77	0
5	3291.93	3402.69	3313.85	0.712	0.720	<0.001	0.81	1
6	3262.49	3393.04	3288.33	0.290	0.295	<0.001	0.79	2
7	3243.66	3393.98	3273.41	0.163	0.170	<0.001	0.82	3
8	3232.30	3402.40	3265.97	0.440	0.449	<0.001	0.83	3

Note. Akaike Information Criterion (AIC); Bayesian Information Criteria (BIC); LRT = Vuong–Lo–Mendell–Rubin Likelihood Ratio Test; BLRT = Bootstrap Likelihood Ratio Test. Size: Number of clusters with fewer than 25 subjects. Bold values indicate the selected model.

**Table 3:** Profile distribution by gender.

	Profile 1	Profile 2	Profile 3	Total
Men	22	78	83	183
	5.7%	20.2%	21.5%	47.4%
Women	15	78	110	203
	3.9%	20.2%	28.5%	52.6%
Total	37	156	193	386
	9.6%	40.4%	50.0%	100.0%

**Table 4:** Means, standard deviations, and partial eta squared (*ηp*^2^) values for each stress dimension across the three profiles.

	Profile 1		Profile 2		Profile 3		Statistical Signification		
Dimensions	*M*	*SD*	*M*	*SD*	*M*	*SD*	*F*_(14,383)_	*p*	*η* ^2^
Teacher Interaction	14.18	5.01	12.58	4.00	10.74	3.57	17.13	<0.001	0.08
Academic Stress	10.97	3.14	10.88	3.11	10.05	3.04	3.68	0.026	0.02
Peer interaction	12.35	3.63	11.19	4.28	9.73	3.69	10.10	<0.001	0.05
Academic Self-Concept	9.43	3.27	9.07	2.99	8.08	2.75	6.55	0.002	0.03
Emotional Stress	14.51	5.86	13.10	4.46	9.50	3.40	43.96	<0.001	0.19
Behavioral Stress	13.08	5.18	10.80	3.48	8.47	2.63	40.83	<0.001	0.18
Physiological Stress	7.97	2.69	7.01	2.76	5.93	2.24	14.35	<0.001	0.07

**Table 5:** Cohen’s d indices for post hoc contrast profiles.

Dimensions	Profile 1–Profile 2	Profile 1–Profile 3	Profile 2–Profile 3
Teacher Interaction	0.38	0.90	0.49
Academic Stress	-	0.30	0.27
Peer interaction	-	0.71	0.37
Academic Self-Concept	-	0.48	0.35
Emotional Stress	-	1.29	0.92
Behavioral Stress	0.59	1.45	0.77
Physiological Stress	-	0.88	0.43

## Data Availability

The data presented in the paper are available upon request from the corresponding author.
